# The diagnostic value of arginase-1 immunostaining in differentiating hepatocellular carcinoma from metastatic carcinoma and cholangiocarcinoma as compared to HepPar-1

**DOI:** 10.1186/1746-1596-7-149

**Published:** 2012-10-30

**Authors:** Nehal A Radwan, Naglaa S Ahmed

**Affiliations:** 1 Pathology Department, Faculty of Medicine, Ain Shams University, Cairo, Egypt

**Keywords:** Arginase-1, HepPar-1, Hepatocellular carcinoma, Metastatic carcinoma, Cholangiocarcinoma

## Abstract

**Background:**

The ability to distinguish hepatocellular carcinoma (HCC) from metastatic carcinoma (MC) involving the liver and cholangiocarcinoma (CC) by immunohistochemistry has been limited by the lack of a reliable positive marker for hepatocellular differentiation. Arginase-1 is a marker for HCC recently described in some literature.

**Aim:**

To examine the immunohistochemical staining of arginase-1 in cases of HCC, MC involving the liver and CC as compared to hepatocyte paraffin antigen -1 (HepPar-1) in an attempt to further define the diagnostic utility of arginase-1 in differentiating these tumors.

**Materials and methods:**

A comparative immunohistochemical study of arginase-1 and HepPar-1expression was performed in 50 HCC cases, 38 cases of MC to the liver from varying sites, 12 cases of CC and 10 specimens of normal liver tissues. The predictive capacity of arginase-1 and HepPar-1 staining was determined using sensitivity, specificity, positive predictive value, and negative predictive value calculations.

**Results:**

All normal liver tissues (no=10), non- neoplastic cirrhotic liver tissues adjacent to HCC (no=42) as well as those adjacent to MC (no= 9) showed diffuse and strong immunostaining for both arginase-1 and HepPar-1. Arginase-1 demonstrated positive immunoreactivity in 42 of 50 (84%) cases of HCC compared with 35 of 50 (70%) for HepPar-1. Only one of 38 (2.6%) cases of MC and one of 12 (8.3%) cases of CC showed positive immunoreactivity for arginase-1. In contrast, HepPar-1 immunoreactivity was detected in 6 of 38 (15.8%) cases of MC and in 2 of 12 (16.7%) cases of CC. Arginase -1 showed a significantly higher sensitivity for HCC diagnosis (84%) compared to HepPar -1(70%) (p=0.016). The specificity of arginase-1 for HCC diagnosis was higher (96%) than that of HepPar -1 (84%); nevertheless, this was not statistically significant (p=0.109). Howerver, the combination of both immunomarkers for the diagnosis of HCC, raised the specificity to 100%.

**Conclusion:**

Arginase-1 immunostaining has a higher sensitivity and specificity than HepPar-1 for HCC diagnosis. Furthermore, the combined use of arginase-1 and HepPar-1 can provide a potentially promising tool to improve the accuracy in distinguishing HCC from metastatic carcinoma and cholangiocarcinoma.

**Virtual slides:**

The virtual slide(s) for this article can be found here: http://www.diagnosticpathology.diagnomx.eu/vs/9991436558072434.

## Introduction

Hepatocellular carcinoma (HCC) is the most common primary liver cancer. The annual number of new cases of HCC worldwide is over one million. Globally, it is the fifth most common cancer and the third leading cause of cancer related death, preceded only by the lung and stomach cancers [1]. The burden of HCC has been increasing in Egypt with a doubling in its incidence rate in the past 10 years [2]. HCC contributes to 14.8% of all cancer mortality in Egypt. It is the second most frequent cancer type in Egyptian males after bladder cancer. The high incidence of HCC in Egypt is attributed to the high prevalence of hepatitis C virus (HCV). HCV is currently the most significant public health problem in Egypt with an overall prevalence of 17.4% in males and 12.2% in females [3].

The distinction of HCC from cholangiocarcinoma and other types of adenocarcinoma metastatic to the liver is a relatively frequent, often challenging dilemma for surgical pathologists and very crucial, as the treatment goal for these tumors are different. Several treatment modalities, including surgical resection, radiofrequency ablation, and transarterial chemoembolization/radioembolization, are available for hepatocellular carcinoma. In contrast, the therapeutic approach for metastatic carcinoma of the liver is often palliative. Thus, correct classification of these tumors is critically important. Although in most cases; the correct diagnosis can be reached through a synthesis of clinical findings, diagnostic imaging modalities and routine evaluation of hematoxylin and eosin (H&E) stained sections, immunohistochemistry may play a very valuable role in clinically atypical and pathologically indeterminate cases, especially challenging because limited tissue is available with core biopsies, so an appropriate selection of antibodies is imperative [4,5].

A limited number of diagnostically useful immunohistochemical markers for identification of hepatocytes in routine surgical pathology practice are available including; hepatocyte paraffin antigen-1(HepPar-1), polyclonal carcinoembryonic antigen (CEA), and CD10, with alfa-fetoprotein (AFP) and glypican-3 labeling some HCCs [6]. However, the utility of each of these markers is limited either by suboptimal sensitivity or difficulty in interpretation [7]. For example, AFP suffers from a low sensitivity of 30% to 50% and its frequent focal staining limiting its utility in small biopsy samples [7-10]. Polyclonal CEA and CD10 can be difficult to interpret because canalicular and diffuse cytoplasmic staining can be difficult to distinguish. Furthermore, the sensitivities of these markers can be low (25% to 50%) in poorly differentiated HCCs for polyclonal CEA and 50% for CD10) [8,10,11]. Over the past decade, HepPar-1, a mitochondrial urea cycle antigen, has been increasingly used as a positive marker for hepatic differentiation. [7,9,12-14]. However, HepPar-1 also suffers from relatively low sensitivity in poorly differentiated hepatocellular carcinomas, where the distinction between hepatocellular carcinoma and adenocarcinoma is most difficult [9,10,13]. In addition, whereas most adenocarcinomas are negative for HepPar-1, gastric, esophageal, and pulmonary adenocarcinomas can demonstrate strong cytoplasmic HepPar-1 staining [7,9,13]. Glypican-3, a heparin sulphate proteoglycan expressed at high levels in HCC, has shown high specificity with suboptimal sensitivity in the diagnosis of HCC when used in isolation as it is well known to be immunoreactive in a wide variety of tumors, including pulmonary squamous cell carcinoma, [15] germ cell tumors, [16] and a subset of gastric adenocarcinomas [17].

A recent literature report characterized a new immunohistochemical marker, arginase-1 as a potential marker of hepatocellular differentiation in both surgical pathology and cytopathology. Arginase exists in 2 isoforms, namely arginase- 1 and arginase-2, both of which are responsible for the hydrolysis of arginine to ornithine and urea in the urea cycle. Of the 2 isoforms, arginase-1 demonstrates high levels of expression within the liver, whereas arginase-2 levels are highest in the kidneys and pancreas and are very low in the liver [6,18]. Arginase-1 is expressed in normal human liver with a high degree of specificity [19]. Specifically, it has been shown by immunohistochemistry to be concentrated in periportal hepatocytes [20].

The current study aims to examine the immunohistochemical staining of arginase-1 in cases of HCC, metastatic carcinoma involving the liver and cholangiocarcinoma as compared to HepPar-1 that is conventially used. This is in an attempt to further define the diagnostic utility of arginase-1 as a reliable positive marker in differentiating these tumors.

## Materials and methods

### Tissue collection

This retrospective study consisted of 50 cases of hepatocellular carcinoma, 38 cases of metastatic carcinoma to the liver, 12 cases of cholangiocarcinoma and 10 specimens of normal liver tissues. All cases were retrieved from the archives of the Pathology Department, Ain Shams University Hospitals during the period between 2006 and 2011. The clinical history, pathology reports and hematoxylin and eosin (H&E) stained slides for all cases were reviewed to confirm the diagnosis. The histologic grade of HCC was established using the World Health Organization criteria [21]. The study was carried out with full local ethics approval.

### Immunohistochemical staining procedure

Four - micron thick sections of the formalin-fixed, paraffin-embedded tissue blocks of all the studied cases were investigated for the presence of a rabbit polyclonal antibody against arginase-1 (H-52: sc 20150, Santa Cruz, Europe) at a dilution 1:200, and a mouse monoclonal antibody against Hep Par-1, (clone OCH1E5, MS-1810-R7, ready to use, Lab vision, CA, USA) with a labelled streptavidin- biotin-peroxidase complex technique. Briefly, Tissue sections were deparaffinized and hydrated in xylene and descending grades of alcohol. After rinsing in PBS, antigen retrieval was performed by treating the tissue sections with citrate buffer, pH 6.0 for 10 min in a 700-W microwave oven. The endogenous peroxidase activity was blocked by incubating the slides in 3% hydrogen peroxide for 5 to 10 min, and then washed in buffer. This is followed by incubation with the primary antibody (arginase-1 or HepPar-1) for 1 h at room temperature. The antibody reaction was detected with the avidin-biotin detection kit using diaminobenzidine (DAB) as chromogen. Sections were counterstained with hematoxylin for 15 seconds before checked under microscope. Normal liver tissues was used as positive control, while negative control was done using the same tissue (normal liver), omitting the primary antibody.

### Immunohistochemical analysis

Only cytoplasmic or cytoplasmic and nuclear reactivity was considered as positive staining for arginase-1. For HepPar-1; positivity was defined as coarsely granular cytoplasmic staining that could not be confused with background staining or endogenous peroxidase staining. Immunoreactivity was semiquantitatively scored by 2 pathologists. The intensity of immunostaining was scored as 0 (no staining), 1+ (weak staining), and 2+ (strong staining). Furthermore, the pattern of staining (diffuse or focal) was recorded. Focal staining was defined as reactivity in <10% of tumor or lesional cells [6].

### Statistical analysis

Statistical analysis was carried out using Statistical Package for Social Science (SPSS 15.0.1 for windows; SPSS Inc, Chicago, IL, 2001). Qualitative variables are expressed as frequencies and percents. Chi square test and Fisher’s exact test was used to examine the relationship between categorical variables. McNemar test was used to assess the statistical significance of the difference between both immunomarkers for the studied cases. The equation used for sensitivity of diagnostic measures was: True positive by the test/(True positive by the test + false negative by the test) and for specificity; the equation was true negative by the test/(true negative by the test + false positive by the test). Positive predictive value (PPV) is calculated as true positive by test/all positive by the test (True positive by the test + False Positive by the test). Negative predictive value (NPV) is calculated as true negative by test/all negative by the test (True negative by the test + false negative by the test) with histologic diagnosis designated as the gold standard.

## Results

### Clinicopathologic features

The fifty cases of HCC were graded as 11 well differentiated, 30 moderately differentiated, and 9 poorly differentiated . All HCC cases are associated with hepatitis C viral (HCV) infection. Forty-two of the 50 HCC cases were surgically resected specimens and had adjacent non-neoplastic liver tissues that revealed cirrhotic liver tissues and 8 were needle core biopsies. Only two cases of HCC were biopsies of metastatic sites (adrenal gland and chest wall) and the remaining were primary to the liver. The 38 cases of metastatic carcinoma to the liver including 25 from colon, 6 from stomach, 1 from gall bladder and 2 each from pancreas, kidney and lung. The non-neoplastic liver tissues adjacent to metastatic carcinomas were detected in 9 cases and revealed no pathological abnormalities.

### Immunohistochemical findings

**Figure 1:**
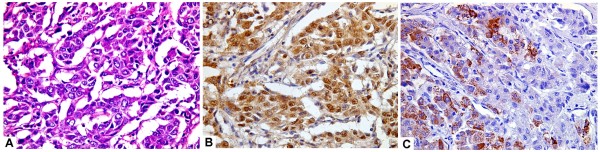
A case of moderately differentiated hepatocellular carcinoma (A, H&E,original magnification x400) with strong and diffuse arginase-1 staining (B; immuoperoxidase, original magnification x400) and focal HepPar-1 immunostaining (C; immuoperoxidase, original magnification x400).

**Figure 2:**
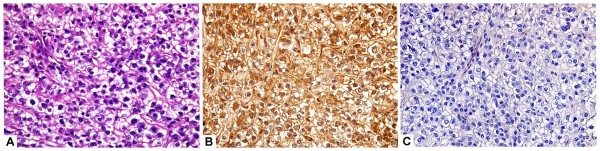
A case of hepatocellular carcinoma (clear cell type) (A, H&E,original magnification x400) with strong and diffuse arginase-1 staining (B; immuoperoxidase, original magnification x400) and negative HepPar-1stainig (C; immuoperoxidase, original magnification x400).

**Figure 3:**
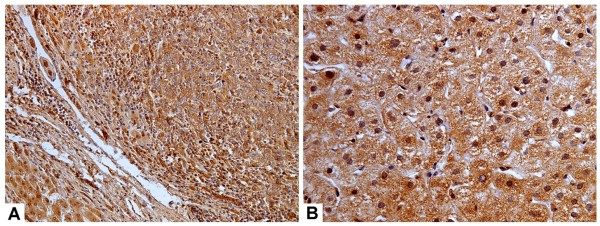
A case of hepatocellular carcinoma (A) with adjacent non-neoplastic liver tissue (B) showed strong and diffuse arginase-1 staining (A,B, immuoperoxidase, original magnification x200, x400).

**Figure 4:**
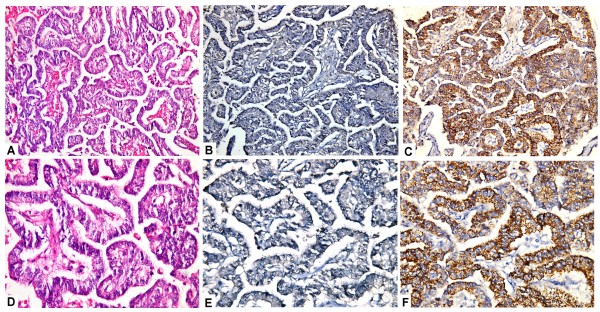
A case of metastatic colonic adenocarcinoma to the liver (A,D; H&E,original magnification x200,x400) showed negative arginase-1 immunostaining (B,E; immuoperoxidase, original magnification x200, x400), and stong and diffuse staining with HepPar-1(C,F; immuoperoxidase, original magnification x200, x400).

**Figure 5:**
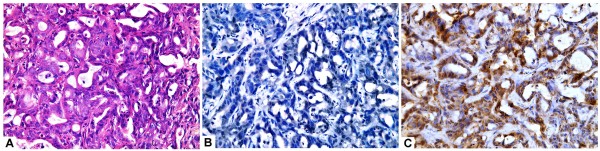
A case of cholangiocarcinoma (A; H&E,original magnification x400) with negative arginase-1 immunostaining (B;immuoperoxidase, original magnification,x400), stong and diffuse staining with HepPar-1 (C; immuoperoxidase, original magnification, x400).

Immunohistochemical expressions of arginase-1 and HepPar-1 in all the studied cases were summarized in Tables 1, 2, 3 and 4 in addition to Figures 1, 2, 3, 4 and 5.

**Table 1:** Clinicopathological features and the expressions of arginase-1 & HepPar-1 in all studied tumorous cases (no=100)

**No**	**Age**	**Sex**	**Histological diagnosis of the tumor**	**Arginase-1 expression**	**HepPar-1 expression**
1	53	M	Well differentiated HCC	2+	2+
2	45	M	Well differentiated HCC	1+	1+
3	48	M	Well differentiated HCC	2+	2+
4	59	F	Well differentiated HCC	2+	2+
5	60	M	Well differentiated HCC	1+	2+
6	49	M	Well differentiated HCC	2+	1+
7	53	F	Well differentiated HCC	2+	1+
8	55	M	Well differentiated HCC	2+	2+
9	48	M	Well differentiated HCC	2+	2+
10	50	F	Well differentiated HCC	1+	1+
11	52	F	Well differentiated HCC	2+	2+
12	49	M	Moderately differentiated HCC	0	0
13	59	M	Moderately differentiated HCC	2+	2+
14	62	M	Moderately differentiated HCC	1+	1+
15	59	M	Moderately differentiated HCC	1+	1+
16	52	F	Moderately differentiated HCC	2+	2+
17	55	M	Moderately differentiated HCC	1+	1+
18	56	F	Moderately differentiated HCC	2+	2+
19	61	M	Moderately differentiated HCC	1+	0
20	60	M	Moderately differentiated HCC	2+	1+
21	59	M	Moderately differentiated HCC	2+	1+
22	51	M	Moderately differentiated HCC	0	0
23	55	F	Moderately differentiated HCC	2+	2+
24	61	M	Moderately differentiated HCC	1+	1+
25	49	M	Moderately differentiated HCC	2+	2+
26	55	M	Moderately differentiated HCC	1+	0
27	54	M	Moderately differentiated HCC	2+	2+
28	59	F	Moderately differentiated HCC	2+	2+
29	62	M	Moderately differentiated HCC	2+	2+
30	61	M	Moderately differentiated HCC	1+	0
31	50	M	Moderately differentiated HCC	1+	1+
32	48	M	Moderately differentiated HCC	2+	1+
33	52	F	Moderately differentiated HCC	2+	2+
34	60	F	Moderately differentiated HCC	1+	0
35	52	M	Moderately differentiated HCC	2+	2+
36	56	M	Moderately differentiated HCC	1+	1+
37	57	M	Moderately differentiated HCC	0	0
38	58	F	Moderately differentiated HCC	2+	2+
39	65	M	Moderately differentiated HCC	2+	2+
40	55	M	Moderately differentiated HCC (metastatic to chest wall)	1+	1+
41	48	M	Moderately differentiated HCC (metastatic to adrenal gland)	1+	0
42	53	M	Poorly differentiated HCC	0	0
43	57	F	Poorly differentiated HCC	1+	0
44	62	M	Poorly differentiated HCC	0	0
45	65	M	Poorly differentiated HCC	1+	1+
46	50	F	Poorly differentiated HCC	1+	0
47	54	M	Poorly differentiated HCC	0	0
48	56	M	Poorly differentiated HCC	0	0
49	52	M	Poorly differentiated HCC	1+	1+
50	60	M	Poorly differentiated HCC	0	0
51	62	F	Metastatic colonic carcinoma	0	0
52	54	M	Metastatic colonic carcinoma	0	0
53	50	F	Metastatic colonic carcinoma	0	0
54	45	F	Metastatic colonic carcinoma	0	0
55	48	M	Metastatic colonic carcinoma	0	0
56	50	F	Metastatic colonic carcinoma	0	1+
57	57	M	Metastatic colonic carcinoma	0	0
58	49	F	Metastatic colonic carcinoma	0	0
59	55	F	Metastatic colonic carcinoma	0	2+
60	50	M	Metastatic colonic carcinoma	0	0
61	52	M	Metastatic colonic carcinoma	0	0
62	60	M	Metastatic colonic carcinoma	0	0
63	51	M	Metastatic colonic carcinoma	0	0
64	55	F	Metastatic colonic carcinoma	0	2+
65	50	M	Metastatic colonic carcinoma	0	0
66	49	F	Metastatic colonic carcinoma	0	0
67	55	F	Metastatic colonic carcinoma	0	0
68	51	F	Metastatic colonic carcinoma	0	0
69	53	M	Metastatic colonic carcinoma	0	0
70	55	M	Metastatic colonic carcinoma	0	0
71	50	M	Metastatic colonic carcinoma	0	0
72	52	M	Metastatic colonic carcinoma	0	0
73	56	F	Metastatic colonic carcinoma	0	0
74	46	M	Metastatic colonic carcinoma	0	0
75	50	M	Metastatic colonic carcinoma	0	0
76	52	M	Metastatic gastric carcinoma	0	2+
77	49	F	Metastatic gastric carcinoma	0	0
78	55	M	Metastatic gastric carcinoma	0	2+
79	56	M	Metastatic gastric carcinoma	0	0
80	50	M	Metastatic gastric carcinoma	0	1+
81	52	F	Metastatic gastric carcinoma	0	0
82	53	M	Metastatic renal cell carcinoma	0	0
83	55	F	Metastatic renal cell carcinoma	0	0
84	61	M	Metastatic pancreatic carcinoma	1+	0
85	62	M	Metastatic pancreatic carcinoma	0	0
86	60	M	Metastatic lung carcinoma	0	0
87	62	F	Metastatic lung carcinoma	0	0
88	55	M	Metastatic gall bladder carcinoma	0	0
89	60	M	Cholagiocarcinoma	1+	0
90	64	M	Cholagiocarcinoma	0	1+
91	59	M	Cholagiocarcinoma	0	2+
92	64	F	Cholagiocarcinoma	0	0
93	62	F	Cholagiocarcinoma	0	0
94	58	M	Cholagiocarcinoma	0	0
95	62	F	Cholagiocarcinoma	0	0
96	60	M	Cholagiocarcinoma	0	0
97	59	F	Cholagiocarcinoma	0	0
98	65	M	Cholagiocarcinoma	0	0
99	62	M	Cholagiocarcinoma	0	0
100	60	M	Cholagiocarcinoma	0	0

M= male, F= female, HCC= hepatocellular carcinoma, HepPar-1=hepatocyte paraffin antigen-1,(0) =no staining, (1+) =weak staining, (2+) =strong staining.

**Table 2:** Summary of immunohistochemical expression of arginase-1and HepPar-1 in all the studied cases

	**Arginase-1**				**HepPar-1**			
	**Negative**	**Positive**		**Total positive**	**Negative**	**Positive**		**Total positive**
	**0 (%)**	**1+(%)**	**2+(%)**	**No (%)**	**0(%)**	**1+(%)**	**2+(%)**	**No (%)**
**Hepatocellular carcinoma (HCC), (no=50):**	8 (16)	19 (38)	23 (46)	42 (84)	15 (30)	16 (32)	19 (38)	35 (70)
well differentiated (no=11)	0 (0)	3 (27.3)	8 (72.7)	11 (100)	0 (0)	4 (36.4)	7 (63.6)	11 (100)
moderately differentiated (no=30)	3 (10)	12 (40)	15 (50)	27 (90)	8 (26.7)	10 (33.3)	12 (40)	22 (73.3)
poorly differentiated (no=9)	5 (55.6)	4 (44.4)	0 (0)	4 (44.4)	7 (77.8)	2 (22.2)	0 (0)	2 (22.2)
**Metastatic carcinoma (MC) (no=38)**	37 (97.4)	1 (2.6)	0 (0)	1 (2.6)	32 (84.2)	2 (5.3)	4 (10.5)	6 (15.8)
Colonic (no=25)	25 (100)	(0)	0 (0)	0 (0)	22 (88)	1 (4)	2 (8)	3 (12)
Gastric (no=6)	6 (100)	0 (0)	0 (0)	0 (0)	3 (50)	1 (16.7)	2 (33.3)	3 (50)
Renal cell carcinoma (no=2)	2 (100)	0 (0)	0 (0)	0 (0)	2 (100)	0 (0)	0 (0)	0 (0)
Pancreas (no=2)	1 (50)	1 (50)	0 (0)	1 (50)	2 (100)	0 (0)	0 (0)	0 (0)
Lung (no=2)	2 (100)	0 (0)	0 (0)	0 (0)	2 (100)	0 (0)	0 (0)	0 (0)
Gall bladder (no=1)	1 (100)	0 (0)	0 (0)	0 (0)	1 (100)	0 (0)	0 (0)	0 (0)
**Cholangiocarcinoma (no=12)**	11 (91.7)	1 (8.3)	0 (0)	1 (8.3)	10 (83.3)	1 (8.3)	1 (8.3)	2 (16.7)
**Non-neoplastic cirrhotic liver tissues adjacent to HCC(no=42)**	0 (0)	0 (0)	42 (100)	42 (100)	0 (0)	0 (0)	42 (100)	42 (100)
**Non-neoplastic liver tissue adjacent to MC (no= 9)**	0 (0)	0 (0)	9 (100)	9 (100)	0 (0)	0 (0)	9 (100)	9 (100)
**Normal liver tissues (no=10)**	0 (0)	0 (0)	10 (100)	10 (100)	0 (0)	0 (0)	10 (100)	10 (100)

HepPar-1=hepatocyte paraffin antigen-1.

**Table 3:** Immunohistochemical expression of arginase-1 and HepPar-1 according to the pattern of staining in all positive cases

	**Arginase-1**			**HepPar-1**		
	**Focal No (%)**	**Diffuse No (%)**	**Total positive**	**Focal No (%)**	**Diffuse No (%)**	**Total positive**
**Hepatocellular carcinoma (HCC), (no=50):**	10 (23.8)	32 (76.2)	42	15 (42.9)	20 (57.1)	35
well differentiated (no=11)	0 (0)	11 (100)	11	1 (9.1)	10 (90.9)	11
moderately differentiated (no=30)	8 (29.6)	19 (70.4)	27	10 (33.3)	12 (40)	22
poorly differentiated (no=9)	2 (50)	2 (50)	4	2 (22.2)	0 (0)	2
**Metastatic carcinoma (MC) (no=38):**	1 (100)	0 (0)	1	1 (16.7)	5 (83.3)	6
Colonic (no=25)	(0)	0 (0)	0	1 (33.3)	2 (66.7)	3
Gastric (no=6)	0 (0)	0 (0)	0	0 (0)	3 (100)	3
Renal cell carcinoma (no=2)	0 (0)	0 (0)	0	0 (0)	0 (0)	0
Pancreas (no=2)	1 (100)	0 (0)	1	0 (0)	0 (0)	0
Lung (no=2)	0 (0)	0 (0)	0	0 (0)	0 (0)	0
Gall bladder (no=1)	0 (0)	0 (0)	0	0 (0)	0 (0)	0
**Cholangiocarcinoma (no=12)**	1 (100)	0 (0)	1	1 (50)	1 (50)	2
**Non-neoplastic cirrhotic liver tissues adjacent to HCC(no=42)**	0 (0)	42 (100)	42	0 (0)	42 (100)	42
**Non-neoplastic liver tissue adjacent to MC (no= 9)**	0 (0)	9 (100)	9	0 (0)	9 (100)	9
**Normal liver tissues (no=10)**	0 (0)	10 (100)	10	0 (0)	10 (100)	10

HepPar-1=hepatocyte paraffin antigen-1.

**Table 4:** Sensitivity, specificity, positive and negative predictive value of arginase-1, HepPar-1 for HCC diagnosis

	**Sensitivity**	**Specificity**	**PPV**	**NPV**
Arginase-1	84%	96%	95.5%	85.7%
HepPar-1	70%	84%	81.4%	73.7%
Arginase-1 or HepPar-1	84%	80%	88.8	83.3%
Arginase-1 and HepPar-1	70%	100%	100%	76.9%

HCC=hepatocellular carcinoma; MC=metastatic carcinoma; CC=cholangiocarcinoma; PPV=positive predictive value; NPV=negative predictive value; HepPar-1=hepatocyte paraffin antigen-1.

All normal liver tissues (no=10), non- neoplastic cirrhotic liver tissues adjacent to HCC (no=42) as well as those adjacent to MC (no= 9) showed diffuse and strong (2+) immunostaining for both arginase-1 and HepPar-1.

Arginase-1 demonstrated positive immunoreactivity in 42 of 50 (84%) cases of HCC compared with 35 of 50 (70%) for HepPar-1. Positive arginase −1 and HepPar-1 expression was present in all 11 cases (100%) of well-differentiated HCC. However; arginase −1 immunostaining was positive in 27 of 30 (90%) cases of moderately differentiated HCC and 4 of 9 (44.4%) cases of poorly differentiated HCC compared with 22 of (30) (73.3%) and 2 of (9) (22.2%) for HepPar-1 respectively . In all studied HCC cases, there were no cases that were positive for HepPar-1 with concurrent negative arginase-1 staining, while 7 HCC cases showed arginase-1 staining but were negative for HepPar-1.

Only one of 38 (2.6%) cases of MC and one of 12 (8.3%) cases of CC showed positive immunoreactivity for arginase-1 and the staining was focal and weak. In contrast, HepPar-1 immunoreactivity was detected in 6 of 38 (15.8%) cases of MC and in 2 of 12 (16.7%) cases of CC.

Among all HCC cases, arginase -1 showed a significantly higher sensitivity for diagnosis of HCC (84%) compared to HepPar -1 (70%) (p=0.016). Within the different grades of HCC; the sensitivities of arginase-1 in well, moderately, and poorly differentiated HCCs are 100%, 90%, and 44.4%, respectively, whereas, in comparison, HepPar-1 demonstrated sensitivities of 100%, 73.3%, and 22.2% for well, moderately, and poorly differentiated tumors, respectively. There was no significant difference between arginase -1 and HepPar- 1 as regards their sensitivities in diagnosis of well or poorly differentiated HCC, while for moderately differentiated HCC cases; arginase -1showed a significantly higher sensitivity than HepPar-1 (p=0.001).

The specificity of arginase-1 for diagnosis of HCC was higher (96%) than that of HepPr −1 (84%); nevertheless, this was not statistically significant (p=0.109). The positive predictive value (PPV) of arginase-1 for distinguishing HCC from MC and CC was higher (95.5%) than that observed with HepPar-1(81.4%). Also, the negative predictive value (NPV) for arginase-1 (85.7%) in distinguishing HCC from MC and CC was better than that of HepPar-1(73.7%). Howerver, the combination of both immunomarkers for the diagnosis of HCC, raised the specificity to 100% as shown in Table 4.

## Discussion

The most commonly encountered differential diagnostic challenge in the liver is HCC versus intrahepatic cholangiocarcinoma or metastatic adenocarcinoma [7]. Some of these diagnostic challenges can be attributed to: a) The liver represent one of the three most common sites of metastasis, b) HCCs may show a variety of histologic patterns, mimicking a wide variety of malignant tumors. In addition, a number of metastatic tumours, notably from the breast, pancreas, kidney and adrenals may mimic the trabecular, liver-like pattern of HCC, c) Cholangiocarcinoma and HCC often share overlapping morphologic appearances, d) Complicating the diagnostic process is that pathologists are frequently asked to handle and diagnose tiny liver needle core biopsies with various biopsy artifacts [9,22]. A limited number of diagnostically useful immunohistochemical markers have been applied in an attempt to differentiate HCC from liver metastases or cholangiocarcinoma including; HepPar-1, polyclonal carcinoembryonic antigen (CEA), and CD10, with alfa-fetoprotein (AFP) and glypican-3 labeling some HCCs [6]. However, the utility of each of these markers has significant diagnostic limitations [7].

A recent study of Hajósi-Kalcakosz et al. [23] published in 2012 investigated enhancer of zeste homologue 2 (EZH2) as a new marker of HCC. They reported that EZH2 was detected by immunohistochemistry in nearly all the investigated HCC, CC, hepatoblastoma, metastatic liver tumors and several other childhood cancers. On the contrary, none of the hepatocellular or biliary adenomas, high grade dysplastic or cirrhotic nodules was positive. Thus, this study concluded that EZH2 is a sensitive and reliable immune marker of hepatocellular carcinoma, compared to non-malignant hepatocellular lesions. However, EZH2 is not specific for HCC, since almost all the investigated malignant liver tumors were positive as well regardless of their histogenesis. Consequently, this marker does not provide help in differentiating the specific histogenesis of liver tumors, but it may well be very useful to differentiate malignant hepatocellular and cholangiocellular tumors from benign tumors and reactive lesions.

Moreover, special stains, such as reticulin stain and CD34 immunostain, are very helpful in the diagnosis of well differentiated HCC. Most studies have shown that absent or decreased reticulin stain or an abnormal reticulin pattern with widened trabeculae is reliable for the diagnosis of well-differentiated HCC. However, Hong et al. [24] reported two cases of well-differentiated HCC with an unusual reticulin staining pattern in their primary biopsies. They suggested that HCC may have diverse reticulin patterns in different portions of the tumor. In a small specimen, such as core biopsy, if only the portion of tumor with well preserved reticulin network is present, the diagnosis can be challenging. Thus, it is important to recognize the presence of different reticulin staining patterns in the evaluation of small biopsies for the diagnosis of HCC.

Arginase-1 has been described in recent literature as a new potential immunohistochemical marker of hepatocellular differentiation [6]. Only few studies investigated arginase −1 expression in HCC and most of these reports performed on fine needle aspiration cytology [5,25,26] with some variation in their interpretations as regards its sensitivity and specificity. Therefore; the primary purpose of the current study was to examine the immunohistochemical staining of arginase-1 in cases of HCC, metastatic carcinoma involving the liver and cholangiocarcinoma as compared to HepPar-1. This is in an attempt to further define its diagnostic utility as a reliable positive marker in differentiating these tumors. HepPar-1 was selected to be compared with this new marker as it is conventially used and has been found to be overrated as a hepatoma marker. The present study examined arginase-1 and HepPar-1 expression in 50 HCC cases, 38 cases of metastatic carcinomas to the liver from varying sites, 12 cases of cholangiocarcinoma and 10 specimens of normal liver tissues. In addition, the non-neoplastic liver tissues adjacent to HCC or metastatic carcinomas were also investigated.

The results revealed that arginase -1 showed a significantly higher overall sensitivity for diagnosis of HCC (84%) compared to HepPar -1 (70%). This confirm the conclusion of the previous studies [5,6,25-27]. It is worth mentioning that there were no cases were positive for HepPar-1, with concurrent negative arginase-1 staining. In addition, arginase-1 showed more diffuse staining in HCC (76.2%) than HepPar-1 (57.1%). This makes interpretation of arginase -1 easier especially in limited liver biopsies.

Furthermore, arginase-1 gave a sensitivity of 100%, 90%, and 44.4% in well, moderately, and poorly differentiated HCCs, respectively, whereas, in comparison, HepPar-1 demonstrated sensitivities of 100%, 73.3%, and 22.2% for well, moderately, and poorly differentiated tumors, respectively. Therefore, arginase-1 showed better sensitivity compared with HepPar-1 in identifying higher grade HCC. This is relatively in accordance with the original paper describing the antibody of Yan et al. [6] who found more marked difference between both immunomakers in poorly differentiated HCCs, in which the sensitivities of arginase-1 and HepPar-1 were 85.7% and 46.4%, respectively. This finding is very useful because one of the most frequent diagnostic challenges facing a pathologist examining liver focal lesion is distinguishing between poorly differentiated HCC from a metastasis, especially in small biopsy specimen. The lower diagnostic sensitivity in our study as compared to that of Yan et al. [6] may be because of the smaller sample size. In contrast, Timek et al. [25] failed to demonstrate a better sensitivity of arginase-1 for higher–grade HCC compared with HepPar-1 and they explained that by the small sampling of the cytologic specimens in the moderately to poorly differentiated HCC category (n =7), limited amount of sample for each case, and patchy/focal staining for arginase-1 in higher-grade HCC.

Moreover, we observed diffuse and strong immunostaining for both arginase-1 and HepPar-1 in the non- neoplastic cirrhotic liver tissues adjacent to HCC as well as those adjacent to MC. This supports the study of Fujiwara et al. [5] and Timek et al. [25] who reported that arginase-1 has no role in distinguishing well-differentiated hepatocellular carcinoma from benign hepatic lesions.

Two very recent studies examined the immunohistochemical expression of L1 cell adhesion molecule (L1CAM) [28] and SOX9 [29] in HCC cases and their adjacent non- neoplastic liver tissues and they reported that immunoreactivity of these markers was significantly increased in substantial proportion of HCC cases compared with their adjacent non- neoplastic liver tissue. Additionally, they suggested that L1CAM expression in HCC was significantly correlated with the advanced tumor progression and was an independent poor prognostic factor for both overall survival and disease-free survival in patients with HCC. Furthermore, SOX9 overexpression in HCC tissues is of predictive value on tumor progression and poor prognosis. Moreover, Schmilovitz-Weiss et al. [30] reported that squamous cellular carcinoma antigen (SCCA) is overexpressed in HCC and it is associated with tumor differentiation, cell proliferation and apoptosis. The results of their study confirm a potential association of negative SCCA expression with other markers of poor outcome in HCC.

In our study, the specificity of arginase-1 for diagnosis of HCC was higher (96%) than that of HepPar -1 (84%). Only one case of pancreatic adenocarcinoma out of 38 (2.6%) cases of MC and one of 12(8.3%) cases of CC showed positive immunoreactivity for arginase-1. However, the staining was focal and weak in these two positive cases. In contrast, HepPar-1 immunoreactivity was detected in 6 of 38 (15.8%) cases of MC (3 from colon and 3 from stomach) and in 2 of 12 (16.7%) cases of CC. Although, neither arginase-1 nor HepPar-1 immunostaining demonstrated 100% diagnostic specificity to distinguish HCC from MC in the liver and CC, our analysis of the combination of both immunomarkers among all studied tumors, raised the diagnostic specificity for HCC to 100% if both showed positive immunostainings. This high specificity of arginase-1 and HepPar-1 combination because the staining patterns of both immunomarkers in adenocarcinomas were mutually exclusive (i.e. arginase-1 - positive adenocarcinomas always lacked HepPar-1 immunoreactivity and vice versa) [5].

These findings are in agreement with the study of Fujiwara et al. [5] which showed that arginase-1 is not entirely specific for hepatic differentiation, as immunoreactivity can be identified in adenocarcinomas, particularly of pancreatic origin. The authors reported that it is not surprising to find a subset of the pancreatic adenocarcinomas included in their analysis demonstrated arginase-1 immunoreactivity. This is because a recent analysis of arginase-1 immunohistochemical expression in rats demonstrated that it was expressed at high levels in the liver and at moderate levels in the pancreas [18]. Moreover, Yan et al. [6] found that only one case of prostatic adenocarcinoma demonstrated arginase-1 immunoreactivity. Of note, their study did not include pancreatic adenocarcinomas in their analysis. In contast, Timek et al. [25] and McKnight et al. [26] reported negativity of arginase-1 in all their cases of MC.

The positive immunostaining of HepPar-1 in our 6 cases of MC (3 from colon and 3 from stomach) was in concordance with the results of Yan et al. [6] who detected HepPar-1 reactivity in 2 colonic adenomas, 8 colonic adenocarcinomas, 2 pulmonary adenocarcinomas, 1 chromophobe RCC, and 9 gastric adenocarcinomas (47.4% of cases). HepPar-1 immunoreactivity in gastric adenocarcinomas is reported in previous studies in which it was expressed in 47% to 83% of gastric cancers [10,13,31]. Moreover, Timek et al. [25] reported that the expression of HepPar-1 in nonhepatocellular tumors is well documented in the literature and they assumed that caution should be taken when using HepPar-1 to confirm a diagnosis of HCC.

In our study, out of 12 cases of CC, only one (8.3%) was positive for arginase-1, while 2 (16.7%) were positive for HepPar-1. This supports the study of Yan et al. as regards arginase -1 reactivity [6]. In addition, Fujiwara et al. [5] reported negative immunoreactivity in all their cases for both immunomarkers. However, the positivity of HepPar-1 in our study is consistent with previous studies [14,22,32]. Shiran et al. [22] claimed that the presence of this occasional positivity should not be surprising considering the common progenitor cell of HCC and CC [14]. On the contrary, Iida et al. [33] concluded that HepPar-1 was rarely but definitely expressed in hilar and peripheral intrahepatic CC, while arginase-1 was expressed at a high rate in both hilar and peripheral intrahepatic CC, irrespective of their histology. They assumed that care should be taken when using arginase-1 as a hepatocyte marker for distinguishing between a poorly differentiated hepatocellular carcinoma and a mass-forming peripheral intrahepatic CC showing the histology of poorly differentiated adenocarcinoma.

One of the important findings in the present study was that arginase-1 showed diffuse and strong nuclear reactivity along with cytoplasmic staining which was observed more in some HCC cases and their adjacent non-neoplastic cirrhotic liver tissues compared with other studied cases. It could be explained as all our HCC cases are associated with HCV. This possible explanation is supported by the findings of Cao et al. [34] who reported that elevated arginase-1 staining is associated with chronic HCV infection as they found that arginase-1 expression was elevated in more than 75% of HCV infected liver samples compared to paired HCC from the same patients (> 33% positive) and to uninfected liver tissues (0% positive). The authors suggested that up-regulated expression of arginase- 1 was associated with HCV infected liver, and to a lesser extent in tumor, but not in uninfected liver. They assumed that an important part of the mechanism whereby HCV regulates hepatocellular growth and survival may be through altering arginine metabolism. However, further studies in large scale are worth-while to confirm these observations.

## Conclusions

In conclusion, the present study demonstrates that arginase-1 immunostaining has a higher sensitivity and specificity than HepPar-1 for HCC diagnosis. Although none of them gives 100% specificity for HCC, but the combined use of arginase-1 and HepPar-1 can provide a potentially promising tool to improve the accuracy in distinguishing HCC from MC and CC. Therefore, from the findings of the current and previous few studies about arginase-1 immunostaining in HCC, we can expect that it will be used as a hepatoma marker in routine surgical pathology practice. However, further prospective studies are recommended to confirm these results.

## Abbreviations

HCC: Hepatocellular carcinoma; MC: Metastatic carcinoma; CC: Cholangiocarcinoma; HCV: Hepatitis C viral; HepPar-1: Hepatocyte paraffin antigen-1; H&E: Hematoxylin and eosin; PPV: Positive predictive value; NPV: Negative predictive value.

## Competing interests

The authors declare that they have no competing interests.

## Authors’ contributions

NAR conceived, designed and coordinated the study, evaluated immunohistochemistry, performed the statistical analysis, carried out photographing and drafted the manuscript. NSA reviewed the histological diagnosis, evaluated immunohistochemistry, participated in the study design and helped to draft the manuscript. All authors read and approved the final manuscript.
